# miR1919–MCD1 Regulation of Debneyol Biosynthesis in *Nicotiana*: Enzyme Organisation, Catalytic Evidence and the Limits of Substrate Channelling

**DOI:** 10.3390/ijms27219951

**Published:** 2026-11-06

**Authors:** Mateusz Labudda

**Affiliations:** Department of Biochemistry and Microbiology, Faculty of Biology and Biotechnology, Warsaw University of Life Sciences—SGGW, Nowoursynowska 159, 02-776 Warsaw, Poland; mateusz_labudda@sggw.edu.pl

**Keywords:** debneyol, phytoalexin, miR1919, MCD1, enzyme organisation, 5-epi-aristolochene, substrate channelling, *Nicotiana*, plant immunity

## Abstract

Debneyol biosynthesis in *Nicotiana* is regulated by the microRNA 1919 (miR1919)–miR1919-targeted cell death factor 1 (MCD1) module, but the mechanisms linking enzyme association to defence-metabolite output remain incompletely resolved. Although MCD1-dependent protein association and catalytic effects have been demonstrated, direct preferential transfer of 5-epi-aristolochene (5-EA) has not. This critical narrative review addresses this evidential gap by distinguishing biochemical pathway reconstruction, protein-interaction assays, enzyme kinetics, transcript responses, metabolite profiles and infection phenotypes, and compares their inferential scope with independent plant metabolic systems. Farnesyl diphosphate is converted via 5-EA and its epoxide to debneyol. In yeast microsomes, MCD1 increased the reported maximum reaction velocity of 5-EA epoxidase (EAE) from 18.07 to 30.24 nM s^−1^, whereas a separate coupled-enzyme assay produced approximately threefold more epoxide. Plant experiments also revealed altered debneyol-to-capsidiol ratios, transcript abundance and infection responses. The novel contribution of this review is a structured appraisal of what each experimental approach establishes and where competing explanations remain unresolved. Direct substrate channelling, transcription-independent branch selection and specific allosteric mechanisms remain unresolved; controlled intermediate-tracing, quantitative interaction and enzyme-abundance studies are needed to discriminate among them.

## 1. Introduction

Infection-induced specialised metabolism provides plants with chemically diverse compounds that can restrict pathogen growth or modify the outcome of colonisation. Phytoalexins are distinguished by their inducible accumulation, but their contribution to resistance must be established for a particular host–pathogen interaction rather than inferred from chemical detection alone. In *Nicotiana attenuata*, challenge with the necrotrophic fungus *Alternaria alternata* elicited sesquiterpenoid metabolism; capsidiol increased from below detection in controls to 50.68 ± 3.10 μg g^−1^ fresh mass three days after inoculation. Silencing genes required for its synthesis increased disease susceptibility, while purified capsidiol inhibited fungal growth in a separate assay [1]. In *Arabidopsis thaliana*, impaired camalexin production increased susceptibility to *Alternaria brassicicola* without producing an equivalent effect against *Botrytis cinerea* under the conditions investigated [2]. These experiments demonstrate that both the chemical identity of a phytoalexin and the infection context determine the meaning of a defence-associated phenotype.

The *Nicotiana* response offers a direct connection between transcriptional activation and chemical defence. Following *A. alternata* infection, an ERF2-like transcription factor activated a gene encoding 5-epi-aristolochene synthase (EAS) and contributed to capsidiol accumulation in *N. attenuata* [1]. This finding identifies a regulator of biosynthetic capacity; the accompanying silencing and antifungal assays link that capacity to a particular biological outcome. It does not follow, however, that increased gene expression determines the route taken by every newly formed intermediate. That problem becomes especially important when several enzymes can consume the same substrate.

The relationship between a biosynthetic pathway and resistance is equally conditional outside Solanaceae. Perturbations of diterpenoid metabolism in rice produced genetically distinguishable disease responses, while interference with glyceollin regulation in soybean altered susceptibility to *Phytophthora sojae* [3,4]. Independent reconstruction of the glyceollin pathway has since provided enzyme-level chemical assignments against which those physiological findings can be interpreted [5]. A resistance phenotype, therefore, acquires biochemical meaning through the combination of a defined intervention, an identifiable metabolic product, and a particular infection experiment, not through metabolite abundance considered in isolation.

The biochemistry of Solanaceae sesquiterpenoids makes the distinction especially consequential. Farnesyl diphosphate (FPP), a precursor of several isoprenoid products, is converted by EAS to 5-epi-aristolochene (5-EA). In elicited *Nicotiana tabacum* cultures, the induction of sesquiterpenoid defence enzymes coincided with reduced squalene synthetase activity, suggesting reorganisation of isoprenoid metabolism in that experimental system [6]. Subsequent precursor studies identified 5-EA as a common intermediate between capsidiol and debneyol [7]. The capsidiol-associated reaction is catalysed by 5-epi-aristolochene hydroxylase (EAH), including the experimentally characterised tobacco CYP71D20 enzyme [8]. The two products therefore share a chemically established precursor, yet their respective formation cannot be inferred from the behaviour of FPP alone.

A biochemical account of the second branch has only recently become possible. In *Nicotiana benthamiana*, Wang and colleagues identified 5-epi-aristolochene epoxidase (EAE) and epoxide hydrolase 1 (EH1), establishing conversion of 5-EA to (R)-5-EA-11,12-epoxide and subsequent formation of debneyol [9]. Their study also linked this route to microRNA 1919 (miR1919) and its target, miR1919-targeted cell death factor 1 (MCD1), which associates with EAS and EAE. Importantly, increased EAS–EAE association, enhanced EAE activity, altered concentrations of branch products, and improved resistance following genetic manipulation were observed in distinct biochemical and physiological experiments. These results identify several levels at which the pathway can be regulated; they do not establish that all measured effects are generated by a single molecular mechanism [9].

In this review, colocalization denotes spatial coincidence without demonstrated molecular binding, whereas physical association refers to experimentally detected protein proximity or co-recovery, with the degree of directness depending on the assay employed. An enzyme complex requires evidence of molecular association, while a metabolic organiser is defined functionally by its capacity to modify enzyme interactions or catalytic behaviour without necessarily constituting a structurally resolved scaffold. A functional metabolon involves coordinated catalytic activity among associated enzymes but does not inherently establish substrate channelling. The latter requires experimental evidence of preferential transfer of the intermediate before equilibration with the accessible metabolite pool.

The MCD1 system thus raises a question that cannot be answered by assigning the pathway enzymes alone. Physical association may increase the effective availability of 5-EA to EAE, MCD1 may alter the catalytic properties of EAE, or changes in transcript and enzyme abundance may contribute to the observed redistribution of products. These explanations are not mutually exclusive. In a membrane-associated cytochrome P450-dependent pathway, local reaction conditions and the supply of reducing equivalents add further variables. Consequently, a change in the debneyol-to-capsidiol ratio is informative about pathway output but cannot serve as a direct measurement of substrate channelling or net metabolic flux [9].

Distinguishing these possibilities requires attention to what each technique actually measures. Protein co-recovery, purified protein binding, enzyme kinetics, and compound identification interrogate different properties of a pathway. Studies of dhurrin biosynthesis and the plant tricarboxylic-acid cycle show how preferential intermediate transfer can be investigated experimentally; work on camalexin demonstrates that catalytic cooperation can be detected in another phytoalexin system [10,11,12,13]. Membrane-associated lignin enzymes, steroidal defence metabolism and structurally studied chalcone synthase–chalcone-isomerase-like (CHS–CHIL) interactions offer additional, non-equivalent comparisons of protein organisation [14,15,16,17]. These systems establish methodological precedents, not conservation of the MCD1 mechanism. Recent rice defence studies provide a further reminder that strong immune phenotypes can arise through regulatory pathways without invoking an analogous enzyme assembly [18].

This review critically evaluates the experimental evidence underlying miR1919–MCD1-dependent debneyol biosynthesis rather than proposing an additional regulatory mechanism. Its principal contribution is to distinguish the causal information provided by pathway reconstruction, protein-interaction assays, enzyme kinetics, transcriptional responses, metabolite profiling and resistance phenotypes. By comparing these distinct experimental readouts with independently characterised phytoalexin and non-phytoalexin pathways, the analysis identifies which mechanistic interpretations are supported, which remain unresolved and which require specifically designed experiments. This evidence-based approach extends the interpretation of the original findings [9] by clarifying the limits of causal inference and establishing experimentally testable criteria for distinguishing enzyme organisation from direct substrate channelling.

## 2. Scope and Critical Appraisal of Experimental Evidence

### 2.1. Literature Scope

This critical narrative review adopts a question-driven appraisal of primary experimental studies, focusing on *Solanaceae* sesquiterpenoid biosynthesis and miR1919–MCD1-dependent regulation. To examine the underlying biological questions, the analysis draws on experimental work in enzyme characterisation, protein interaction, gene and small-RNA regulation, metabolite profiling and pathogen infection. Camalexin, diterpenoid phytoalexins, glyceollins and stilbenes provide comparisons of chemically distinct defence pathways, whereas cyanogenic, flavonoid and steroidal-metabolite systems illustrate experimental approaches to protein organisation and reaction coupling. The purpose of these comparisons is mechanistic discrimination, not the assumption that different plant species share one conserved metabolon architecture.

### 2.2. Appraisal of Mechanistic Evidence

The discussion places particular weight on experiments in which a defined intervention was linked to an identified reaction product, protein interaction, catalytic measurement or infection phenotype. Enzyme assignments are considered in relation to substrate and product identification; interaction experiments in relation to assay design and protein expression context; and resistance experiments in relation to host species, pathogen, and genotype. Evidence from dhurrin biosynthesis and *Solanum* steroidal metabolism provides independent tests of intermediate accessibility and protein organisation, respectively, without establishing the architecture of the MCD1-associated pathway [9,10,11,12,15,16].

The strength of an inference depends on the variable measured. Protein co-recovery can establish an association in a defined preparation, whereas a change in enzyme activity establishes functional modulation without identifying its structural basis. Similarly, accumulation of a metabolic end product reflects the combined effects of synthesis, turnover and transport, rather than an instantaneous biosynthetic flux. Preferential transfer of a newly formed intermediate must be distinguished experimentally from conversion after equilibration with an accessible substrate pool [9,10,11,12,13]. The following sections therefore move from the reaction chemistry itself to protein-level regulation and physiological consequences. Comparisons with other metabolic systems are introduced only where they help to resolve what can be concluded from a particular method or genetic intervention.

## 3. Sesquiterpenoid Reaction Chemistry and MCD1-Associated Organisation

### 3.1. The Shared 5-Epi-Aristolochene Precursor

The shared biochemical precursor of capsidiol and debneyol raises two related but distinct questions: how is carbon supplied to sesquiterpenoid defence, and how is the newly formed 5-EA used in downstream reactions? In elicited *N. tabacum* cultures, reduced squalene synthetase activity accompanied induction of sesquiterpenoid-biosynthetic enzymes [6]. Precursor experiments separately identified 5-EA in the route to both phytoalexins [7]. The first observation concerns elicitation-related enzyme regulation; the second establishes a chemical branch point. Neither constitutes a direct measurement of whole-plant FPP allocation or a demonstration that MCD1 controls sterol biosynthesis [6,7,9]. EAS cyclises FPP to 5-EA, after which hydroxylation and epoxidation provide chemically different routes to capsidiol and debneyol. This branch point makes the relative availability of enzymes and intermediate substrate biologically relevant. Nevertheless, even marked differences in final product concentration cannot establish the proportion of newly synthesised 5-EA entering either pathway without information about reaction rates or product turnover [7,8,9].

### 3.2. Capsidiol-Associated Hydroxylation and Defence Phenotypes

The capsidiol branch depends on hydroxylation of 5-EA. Tobacco CYP71D20 was functionally characterised as a 5-epi-aristolochene-1,3-dihydroxylase using heterologous expression and microsomal assays [8]. EAH must therefore be distinguished from EAE, which catalyses a chemically different transformation in the debneyol branch. Their ability to use a related precursor does not imply identical catalytic chemistry or demonstrate that either enzyme receives 5-EA through a protected transfer route [8,9].

Evidence that capsidiol contributes to immunity is similarly specific to the experimental host and pathogen. In *N. attenuata*, interference with EAS- or EAH-associated genes reduced capsidiol-linked outputs and modified susceptibility to *A. alternata*, while the antimicrobial activity of purified capsidiol was assessed separately [1]. In *Nicotiana benthamiana*, Shibata and colleagues examined the ethylene-associated induction of *NbEAS* and *NbEAH*, and virus-induced gene silencing, in the context of age-related resistance to *Phytophthora infestans* [19]. These investigations support a functional contribution from capsidiol biosynthesis but do not measure a privileged EAS–EAH intermediate-transfer route or establish the action of MCD1 [1,19].

Transcriptional regulation supplies a further, independent level of control. NaWRKY70-dependent responses in *N. attenuata* involved phytohormonal and capsidiol-related defence readouts during *A. alternata* infection [20,21], whereas NaWRKY3 was implicated in a wider brown-spot resistance network [22]. These results underscore why changes in final phytoalexin accumulation need not arise exclusively from protein assembly. They also prevent the mechanistic analysis of MCD1 from becoming detached from the transcriptional networks that underlie tobacco metabolism.

### 3.3. Debneyol Biosynthesis: Reaction Assignments and Reconstruction

**Figure 1:**
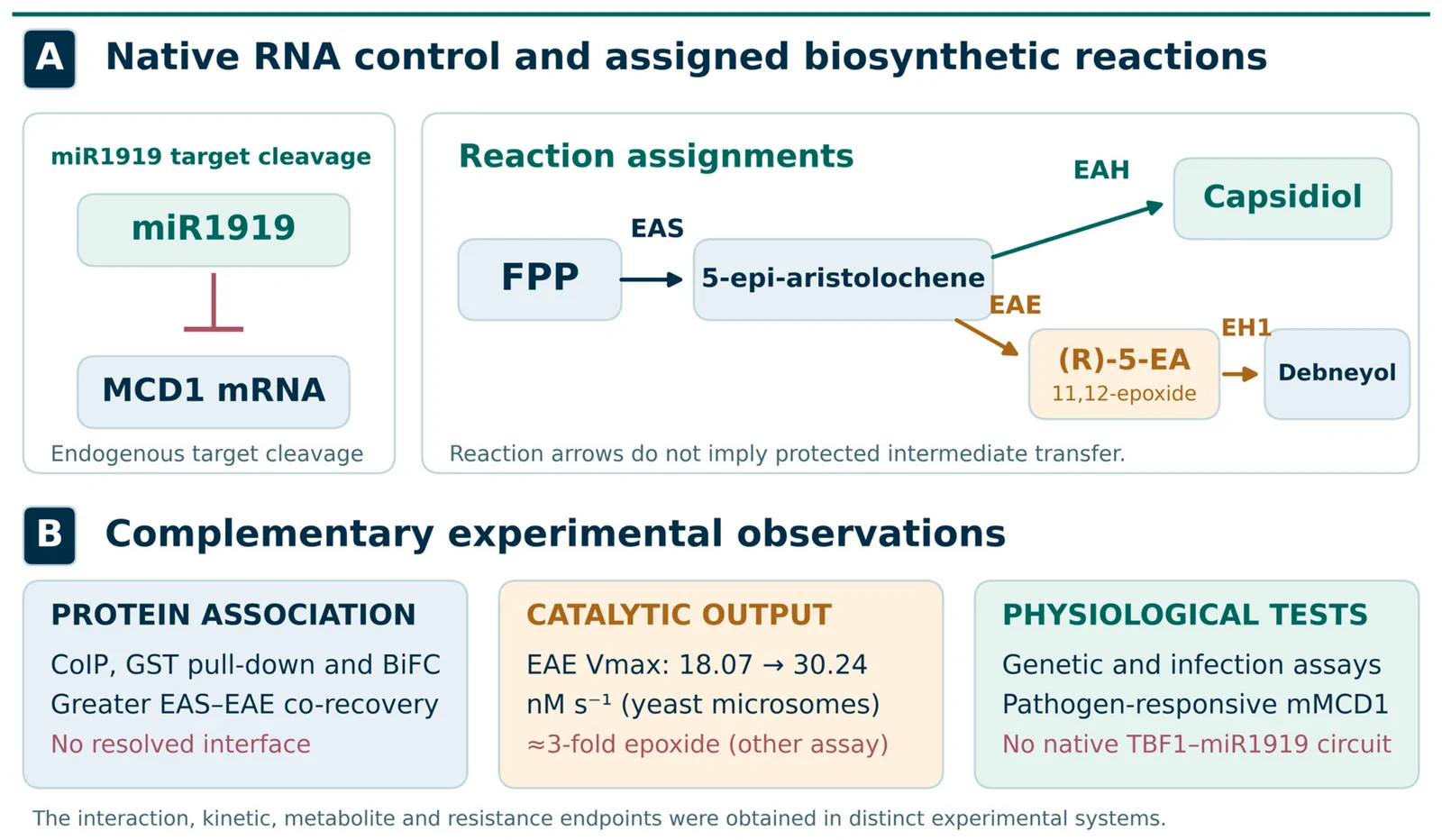
Native miR1919 regulation, assigned sesquiterpenoid reactions and independent MCD1-associated experiments. (**A**) Native miR1919-mediated cleavage of MCD1 transcripts is shown separately from the established chemical sequence. Farnesyl diphosphate (FPP) is converted by 5-epi-aristolochene synthase (EAS) to 5-epi-aristolochene (5-EA); 5-epi-aristolochene hydroxylase (EAH) acts in the capsidiol branch, whereas 5-epi-aristolochene epoxidase (EAE) and epoxide hydrolase 1 (EH1) catalyse steps towards debneyol through (R)-5-EA-11,12-epoxide [7,8,9]. (**B**) Co-immunoprecipitation (CoIP), glutathione S-transferase (GST) pull-down and bimolecular fluorescence complementation (BiFC) support association under their respective conditions. In separate functional assays, EAE V_max_ increased from 18.07 to 30.24 nM s^−1^ and EAS–EAE epoxide production increased approximately threefold with MCD1. The pathogen-responsive TL1-binding factor 1 (TBF1) promoter, upstream open reading frames (uORFs) and miR1919-resistant MCD1 construct were used for engineering rather than observed as an endogenous regulatory circuit [9]. All elements are schematic; arrows denote assigned reactions, not protected substrate transfer.

Debneyol formation was resolved by identifying and reconstructing a three-enzyme pathway. EAS generates 5-EA from FPP; EAE catalyses the formation of (R)-5-EA-11,12-epoxide; and EH1 converts the epoxide into debneyol. The enzyme assignments were supported by heterologous expression, product analysis by gas chromatography–mass spectrometry (GC–MS), comparison with chemically synthesised standards, purified enzyme reactions, and genetic perturbations [9]. Taken as a whole, these experiments establish the principal chemical sequence without requiring a specific model for movement of the intermediates between catalysts. This chemical distinction sets the boundary for the protein-level interpretation. The hydroxylation leading to capsidiol and the epoxidation leading to debneyol compete conceptually for the 5-EA intermediate, but a measured metabolite ratio does not necessarily reflect the relative instantaneous flux through either branch. Figure 1 separates the assigned reactions from both endogenous miR1919 regulation and observations obtained in distinct MCD1-related assays. None of the reaction arrows denotes an enclosed molecular conduit or MCD1-dependent suppression of squalene synthesis [6,7,8,9].

### 3.4. MCD1 Interactions and Localisation: Assay-Specific Evidence

The physical evidence for MCD1-associated organisation comes from complementary interaction assays. Co-immunoprecipitation (CoIP) in transiently expressing *N. benthamiana* leaves recovered MCD1 with EAS1.1, EAS5.1 and EAE. Glutathione S-transferase (GST) pull-down experiments with purified fusion proteins provided additional evidence of binding in defined in vitro preparations. In plant co-expression experiments, MCD1 increased detectable EAS–EAE association and reduced EAS–EAH co-recovery when both downstream enzymes were present [9]. The association response was further examined across MCD1 mutant and expression backgrounds, while bimolecular fluorescence complementation (BiFC) provided a spatially informative EAS–EAE proximity readout. These findings support MCD1-dependent reorganisation of detectable protein associations. They do not determine native stoichiometry or atomic interfaces, and the high concentrations achieved by transient expression remain a relevant consideration when interpreting interaction strength.

EH1 was also co-recovered with components of the proposed pathway-associated assembly [9]. This suggests that the terminal hydrolytic reaction may occur within the same protein neighbourhood, but CoIP cannot establish direct binding between every partner or protected transfer of epoxide to EH1. EAE is a membrane-associated cytochrome P450, whose activity depends on appropriate electron supply and membrane conditions. Experiments in other P450-dependent systems show that redox partners and local membrane composition can modulate catalytic output independently of intermediate channelling [23,24,25,26,27]. They do not establish that either mechanism accounts for the MCD1–EAE interaction.

### 3.5. MCD1-Dependent Activity, Products and Measured Limits

The most direct kinetic comparison concerns EAE assayed in yeast microsomes containing cytochrome P450 reductase. In the presence of MCD1, the reported maximum reaction velocity (V_max_) increased from 18.07 to 30.24 nM s^−1^ (approximately 1.7-fold), while immunoblotting indicated comparable EAE protein abundance [9]. However, the original study did not report normalisation of V_max_ to the concentration of catalytically active EAE. Consequently, the observed increase cannot be assigned specifically to a higher catalytic turnover number (k_cat_). It may also reflect changes in the proportion of functionally active EAE or its effective microsomal environment, including membrane organisation and electron-transfer efficiency. In a separate coupled EAS–EAE assay, MCD1 increased epoxide production approximately threefold [9]. Because these experiments differed in substrate supply, enzyme composition, and measured output, their effect sizes cannot be attributed to a single kinetic mechanism. Collectively, the findings support MCD1-dependent functional modulation of EAE, while the underlying molecular mechanism remains unresolved.

Metabolite partitioning presents a related but distinct regulatory problem. In transiently expressing *N. benthamiana* leaves containing EAE and EAH, MCD1 increased the ratio of debneyol to capsidiol plus capsidiol 3-acetate (3-Ac-capsidiol). In the corresponding samples, EAH transcript abundance increased approximately 2.3-fold and EAE approximately 1.6-fold [9]. In a separate experimental configuration involving elevated EAS expression, MCD1-deficient plants exhibited a lower debneyol-related product ratio than wild-type plants, despite broadly comparable total concentrations of the measured 5-EA-derived products [9]. Changes in EAS, EAE, and EH1 transcript abundance were also documented under other MCD1-expression conditions [9]. Although these observations indicate that pathway output and transcriptional regulation can change concurrently, they do not permit quantitative separation of the contributions of transcript abundance, enzyme abundance, and protein association. Furthermore, comparable EAE protein levels in the yeast microsomal kinetic assay cannot substitute for matched protein-abundance measurements in the plant metabolite experiments. The relative contributions of these regulatory processes therefore remain experimentally inseparable under the reported conditions. Independent interaction and kinetic assays nevertheless support an MCD1-dependent functional effect without establishing its exclusive contribution to metabolite redistribution.

Distinguishing these possibilities calls for an experiment designed around intermediate accessibility. A reconstituted EAS–EAE system could be challenged with isotopically distinguishable newly formed and externally supplied 5-EA; a competing intermediate sink would test whether EAE preferentially consumes the nascent substrate before equilibration. Relevant conceptual precedents exist in the literature on dhurrin and the plant tricarboxylic acid cycle [10,11,12]. Quantitative interaction measurements at controlled protein abundance, together with interface-directed mutations retaining intrinsic enzyme activity, would separately test recruitment and catalytic modulation. These are proposed discriminating measurements, not results already obtained for MCD1. Direct protected EAS-to-EAE transfer, EAE-to-EH1 transfer, and a resolved MCD1-induced allosteric transition remain unmeasured [9,17].

The relationship between debneyol biosynthesis and pathogen resistance must be evaluated across distinct experimental interventions. Silencing EAS, EAE or EH1 increased the susceptibility of *N. benthamiana* to *B. cinerea* and TNV-AC, supporting a contribution from the biosynthetic pathway under the tested conditions [9]. Exogenous application of purified debneyol provided complementary evidence of defence-related chemical activity but did not establish the concentrations or spatial distribution of endogenously synthesised debneyol at infection sites. Separately, MCD1 loss- and gain-of-function experiments revealed altered responses to fungal, viral and bacterial challenges, while genetic assays demonstrated MCD1-associated cell death [9]. The reported requirement for EAS in MCD1-triggered cell death indicates functional coupling between sesquiterpenoid metabolism and this phenotype; it does not establish that debneyol alone mediates cell death or pathogen resistance. Consequently, the available evidence does not permit quantitative separation of phytoalexin-associated antimicrobial effects from other MCD1-associated immune and cell-death responses. These processes may contribute jointly to the observed resistance phenotypes, but their individual causal contributions remain unresolved.

## 4. Related Phytoalexin Systems: Chemically Distinct Lessons

### 4.1. Camalexin: Linked Reaction Chemistry and Infection Outcomes

The nearest conceptual parallel to the *Nicotiana* system is not a chemically homologous pathway but another phytoalexin pathway in which both catalysis and protein cooperation have been studied. In *A. thaliana*, PAD3 genetics, CYP71A13 characterisation and reconstruction with a defined complement of cytochrome P450 enzymes progressively established reactions required for camalexin formation [28,29,30]. These experiments assign chemistry based on independent genetic and enzymatic evidence. They do not imply that the Solanaceae EAS–EAE sequence shares an enzyme architecture with tryptophan-derived camalexin.

Mucha and colleagues then investigated a camalexin-associated metabolon using interaction assays and microsomal kinetics [13]. Co-expression of CYP71A13 reduced the apparent K_m_ reported for CYP79B2 with tryptophan from 17.5 ± 1.9 to 6.9 ± 0.9 μM in the tested preparation. That kinetic change shows why enzyme cooperation can be examined without assuming an atomic allosteric interface or a sealed conduit for the intermediate. It provides a relevant comparison for interpreting MCD1-dependent EAE activity, not proof of a shared physical mechanism [9,13].

Even a chemically established defence metabolite cannot be assigned a context-free resistance function. In *pad3* mutant *Arabidopsis*, Thomma and colleagues observed increased susceptibility to *A. brassicicola* but not *to B. cinerea* in the comparison tested [2]. A separate study implicated camalexin in partial resistance to *Plasmodiophora brassicae* [31], while direct assays of camalexin action examined antimicrobial effects in relation to pathogen behaviour [32]. The relevant unit of evidence is therefore the compound, genetic background and pathogen considered together.

These infection studies illustrate a fundamental asymmetry in the interpretation of defensive metabolism. Gene disruption can demonstrate that a biosynthetic function is required for a particular resistance phenotype, whereas an in vitro antimicrobial assay establishes chemical activity under its own exposure conditions. Neither observation alone demonstrates that endogenous concentrations at the infection site are sufficient to account for every aspect of resistance [2,31,32]. Camalexin therefore offers a useful physiological comparator without being a surrogate for the regulation or intracellular organisation of the debneyol pathway.

### 4.2. Rice Diterpenoids: Genetic Specificity Without Inferred Scaffolding

A comparable limitation emerges from rice (*Oryza sativa*), whose diterpenoid phytoalexins are chemically distinct from tobacco sesquiterpenoids. Reverse-genetic analysis of *OsCPS4*-associated biosynthesis provided evidence for specific physiological roles [33]; a subsequent comparison of *OsCPS2*- and *OsCPS4*-dependent metabolites linked different branches to distinct pathogen outcomes [3]. These are pathway-level, infection-dependent conclusions, not evidence of protected intermediate transfer among a conserved set of enzymes.

More recent rice experiments linked the OsWRKY10–OsVQ8 regulatory module to changes in diterpenoid phytoalexin accumulation and resistance to defined pathogens [18]. The study provides direct regulatory and physiological data, but the effects of a transcriptional regulator on several defence readouts cannot be reduced to the isolated action of a single metabolite. Causal interpretation requires that the genotype, chemical measurements, and infection endpoint remain paired.

### 4.3. Soybean Glyceollins: Enzymatic Reconstruction and Causal Defence Tests

In soybean (*Glycine max*), recent biosynthetic reconstructions have assigned enzymes involved in glyceollin formation and reproduced steps of the route in heterologous settings [5,34]. This work demonstrates the value of chemically verified enzyme assignments, especially when multiple products arise from related isoflavonoid precursors. These studies establish the biochemical basis of glyceollin biosynthesis but provide no evidence for an MCD1-like regulatory mechanism or equivalent enzyme organisation. The comparison is most informative when reaction identity and defence phenotype are treated as separate claims.

In soybean, RNA interference perturbation of isoflavonoid biosynthesis altered race-specific resistance and hypersensitive cell death outcomes following *P. sojae* challenge [35]. Independent experiments linked suppression of glyceollin formation with increased susceptibility to *P. sojae* and *Macrophomina phaseolina* in the tested tissues [36]. Genetic, promoter-binding and infection analyses subsequently implicated GmMYB29A2 in the regulation of glyceollin I and soybean resistance [4]. The interpretation must remain pathway-specific: changing an upstream isoflavonoid reaction can also alter multiple downstream metabolites.

Transgenic soybean hairy roots provide a further intervention-based example. The introduction of stilbene pathway genes or the manipulation of glyceollin biosynthesis altered defence metabolite accumulation and root necrosis after fungal challenge in experiments by Zernova and colleagues [37]. These measurements demonstrate that modifying chemical defence capacity can alter a defined disease phenotype; they do not establish the assembly of a phytoalexin metabolon.

### 4.4. Grapevine Stilbenes: Transcriptional and Small-RNA Regulation

Stilbene-based defence supplies a different comparison, one in which expression engineering and host context are prominent. Transgenic expression of a foreign stilbene-forming reaction modified resistance in a recipient species [38]; inducible stilbene synthase constructs were later tested in grapevine rootstock [39], and *VpSTS29/STS2* manipulation was linked with fungal tolerance [40]. Population analyses also associate stilbenoid accumulation and transcriptional responses with resistance to *Plasmopara viticola* [41]. The intervention studies and population correlations differ in causal resolution; none establishes enzyme assembly as the mediator of the reported phenotypes. Direct small-RNA experiments extend this comparison. In grapevine, miR827, which targets *VqMYB14*, was connected to stilbene production and immune susceptibility [42]. A separate analysis of the miR3398–*VqMYB15*-linked target regulation measured stilbene-related outcomes in *Vitis quinquangularis* [43]. These studies establish RNA-dependent regulation of defence metabolism through transcription-factor targets, not a second example of miRNA-controlled protein organisation homologous to MCD1.

### 4.5. Cross-System Synthesis: Regulatory Differences and Evidential Boundaries

The four phytoalexin systems illustrate distinct levels of regulation rather than conserved molecular architecture. In Arabidopsis, camalexin biosynthesis provides experimental evidence of enzyme association and catalytic cooperation but does not establish an MCD1-like organiser or protected intermediate transfer [13,28,29,30]. In rice, genetic perturbations of diterpenoid biosynthetic branches and the OsWRKY10–OsVQ8 regulatory module link pathway regulation to pathogen-dependent resistance without demonstrating metabolon assembly [3,18,33]. Soybean studies combine biochemical reconstruction of glyceollin biosynthesis with genetic and transcriptional interventions affecting defence phenotypes but provide no corresponding evidence of an MCD1-like enzyme organiser [4,5,34,35,36,37]. In grapevine, stilbene synthase manipulation and miR827–VqMYB14- and miR3398–VqMYB15-associated regulation demonstrate expression-dependent and small-RNA-mediated control of defence metabolism rather than direct microRNA-dependent enzyme recruitment [38,39,40,41,42,43]. Accordingly, these systems offer complementary experimental approaches to establishing reaction chemistry, regulatory causality and physiological relevance. Their mechanisms cannot be transferred directly to *Nicotiana*, where physical association, catalytic modulation and preferential intermediate transfer must be evaluated as separate biochemical propositions [9,13].

## 5. Methodological Analogies from Non-Phytoalexin Metabolism

### 5.1. Dhurrin: Intermediate Accessibility and Enzyme Coupling

The cyanogenic glucoside dhurrin offers a stringent test of intermediate accessibility, although it is not a phytoalexin. In microsomes from *Sorghum bicolor*, Møller and Conn compared internally generated pathway intermediates with compounds supplied from outside the system [10]. They observed substantial preferential utilisation of certain intermediates, whereas another, *p*-hydroxyphenylacetaldoxime, exchanged with the surrounding pool. Thus, even consecutive steps within a single pathway need not exhibit identical transfer behaviour. Subsequent reconstitution of the dhurrin-producing enzyme ensemble examined dynamic association and product formation through complementary biochemical and biophysical methods [11]. Together, these studies show what an informative channelling experiment must discriminate; they do not establish a corresponding transfer mechanism for EAS–EAE. Engineering studies show that completeness matters independently of protein proximity. Expression of a reconstructed dhurrin biosynthetic pathway in *Arabidopsis* led to measurable biochemical outcomes [44]. Disruption of the sorghum glucosyltransferase UGT85B1 altered the products and consequences of incomplete dhurrin biosynthesis [45]. Neither metabolite phenotype, in isolation, identifies a privileged microscopic transfer route.

### 5.2. Membrane-Associated P450 Enzymes and Redox Partners

Different specialised metabolite pathways exhibit experimentally distinct forms of protein association at or near the endoplasmic reticulum (ER). In soybean, interaction and localisation experiments implicated isoflavone synthase and cinnamate 4-hydroxylase in the recruitment of other isoflavonoid enzymes [46]. The reported interaction patterns depended on the enzyme isoform, whereas proteins recovered by affinity purification were candidate members of an assembly rather than verified direct partners. Comparisons of flavonoid enzymes in snapdragon and torenia revealed distinct interaction networks between the two species [47]. Such variation argues against transferring a protein-contact map from one biosynthetic system to another; it also helps explain why physical assembly must be assessed in the relevant tissue, genotype and expression context.

Colocalisation and functional coupling of phenylalanine ammonia-lyase with cinnamate 4-hydroxylase were examined experimentally in an early phenylpropanoid study [48]. That work supplies a historical reference point for pathway organisation, but its results cannot be transferred as evidence for protected 5-EA transfer within the MCD1-associated assembly.

Electron transfer provides a second explanation for altered output from membrane-associated cytochrome P450 enzymes. In *Arabidopsis*, cytochrome b5 was experimentally required for efficient syringyl lignin biosynthesis, although subsequent work showed that b5-dependent activities differ across plant lineages [23,24]. Tea flavonoid experiments are especially instructive: a cytochrome b5 partner increased the production of B-ring-trihydroxylated flavan-3-ols, while substitutions of conserved haem-coordinating residues impaired the catalytic contribution without abolishing the tested interaction [25]. These experiments separate protein binding from redox-supported catalysis. They do not identify a cytochrome b5 requirement for EAE, but they prevent an increase in P450 output from being interpreted automatically as substrate channelling.

Biophysical studies further show how membrane context can influence the P450 redox machinery. Bavishi and colleagues directly observed conformational heterogeneity of P450 oxidoreductase under varying membrane-like and ionic conditions [26]. Jensen and colleagues connected ligand-dependent modulation of oxidoreductase behaviour with biassed P450-mediated product formation [27]. The results are relevant to the interpretation of membrane-enzyme assays, but neither demonstrates two-dimensional diffusion of 5-EA nor does it demonstrate allosteric regulation of EAE by MCD1.

### 5.3. Steroidal Defence and CHS–CHIL: Different Forms of Organisation

The *Solanum* steroidal-metabolite literature demonstrates why the term ‘organiser’ does not imply biochemical inactivity. Jozwiak and colleagues showed that the cellulose-synthase-like protein GAME15 contributes to steroidal glycoalkaloid biosynthesis through both cholesterol glucuronosyltransferase activity and an organisational role [15]. Boccia and colleagues identified a related class of pathway-associated proteins required for the production of steroidal defence metabolites; genetic disruption in *Solanum nigrum* depleted both steroidal glycoalkaloids and saponins, helping to establish an insect-defence role for the latter [16]. These are direct biochemical and genetic findings in steroid-derived pathways. They illustrate the diversity of specialised-metabolite organisation, not a conserved architecture, catalytic function or evolutionary equivalence with *Nicotiana* MCD1.

A complementary example concerns CHS and chalcone-isomerase-like proteins (CHILs). Waki and colleagues combined interaction experiments with product analysis and found that CHIL can redirect the product specificity of the relatively promiscuous CHS reaction [49]. Subsequent structural and kinetic studies investigated the dynamic behaviour of CHS–CHIL systems, including an enzyme pair from *Physcomitrium patens*, and defined partner-dependent changes in catalysis [17,50]. These observations extend beyond colocalization by associating interaction with measured chemistry. Yet CHS is a soluble type III polyketide synthase, whereas EAE is an ER-associated P450 that depends on electron transfer; no equivalent conformational or structural mechanism has been demonstrated for its regulation by MCD1 [9,17].

These comparisons do not enlarge the proposed MCD1 assembly or establish its conservation beyond *Nicotiana*. Their value lies in demonstrating that protein association, catalytic activity, redox dependence, and intermediate accessibility can be examined as distinct properties of specialised metabolic pathways. Table 1 brings together the corresponding observations and the mechanistic conclusions that cannot be transferred across pathways.

## 6. Regulatory RNA, Engineered Expression and Resistance

### 6.1. The Native miR1919–MCD1 Relationship in Context

The endogenous RNA layer of the debneyol system is supported by miRNA target mapping and genetic perturbation, not by an experimentally measured bistable ‘switch’. In *N. benthamiana*, 5′ rapid amplification of cDNA ends (5′ RACE), manipulation of miR1919, cleavage-resistant MCD1 variants, and associated phenotypes linked miR1919 to MCD1 transcript cleavage [9]. These experiments establish sequence-dependent post-transcriptional control. They do not identify a regulatory threshold, demonstrate hysteresis or quantify a time-dependent state transition. The appropriate question is how established RNA-mediated control contributes to the protein concentrations and enzymatic outputs measured under defined conditions.

Plant small RNAs regulate defence chemistry through several non-equivalent routes. In *A. thaliana*, miR393-associated signalling altered the relative accumulation of camalexin and glucosinolate-related metabolites [51]. A separate study linked microRNA-dependent regulation to camalexin and resistance to the green peach aphid, *Myzus persicae* [52]. In *O. sativa*, perturbation of *OsDCL1a* compromised the biosynthesis of diterpenoid phytoalexins and resistance [53]. Each study connects a defined RNA-regulatory intervention with an experimentally measured chemical or physiological output. None demonstrates an MCD1-like interaction between the regulatory target and a biosynthetic enzyme assembly; that mechanistic distinction is central to interpreting the *Nicotiana* pathway.

An *Arabidopsis* microRNA was also linked to an indole-3-acetaldoxime-related metabolic branch through repression of *SUPERROOT1*; the experimentally emphasised outcome was auxin biosynthesis, not phytoalexin scaffold assembly [54]. By comparison, the grapevine miR827a–*VqMYB14* [42] and miR3398–*VqMYB15* [43] studies measured defence-associated stilbenoid outputs. Together, these experiments show regulatory diversity without implying a conserved mechanism of protein organisation.

### 6.2. Endogenous RNA Regulation Versus TBF1–uORF Engineering

Translation control provides a distinct experimental route for engineering disease resistance. The transcription factor TL1-binding factor 1 (TBF1) participates in defence-associated changes in growth-related gene expression, and upstream open reading frames (uORFs) were subsequently used to design pathogen-responsive translation of defence constructs [55,56]. An additional study examined modification of a TBF1-related uORF in rice [57]. These investigations establish the feasibility of conditional expression strategies, not an endogenous regulatory connection between TBF1 and miR1919.

In the *Nicotiana* study, an miR1919-resistant, Myc-tagged MCD1 construct was placed under the control of a pathogen-responsive TBF1 promoter and uORF-based translational control [9]. The engineered *N. benthamiana* lines showed improved resistance to TNV-AC, *B. cinerea* and *Pseudomonas syringae* pv. *tabaci*, without the conspicuous developmental abnormalities associated with sustained MCD1 expression under the reported growth conditions. The regulatory architecture was deliberately engineered; it should not be conflated with native miR1919-mediated repression. Its agricultural value will depend on the durability of resistance, maintenance of growth and reproductive performance, and responses under conditions not examined in the original experiments.

### 6.3. Engineering Defence-Metabolite Pathways and Fitness Consequences

Early transgenic work established that introduction of a foreign phytoalexin biosynthetic activity can alter the disease resistance of a recipient plant [38]. Inducible stilbene synthase expression was subsequently examined in a grapevine-derived genetic background [39], and targeted studies of *VpSTS29/STS2* provided another link between stilbene biosynthesis and fungal tolerance [40]. The interpretation of each intervention depends on the expressed construct, tissue and infection system; none implies MCD1-dependent enzyme assembly.

Metabolite-pathway manipulation can also affect disease outcomes without invoking a scaffold. *Arabidopsis CYP83A1* mutation was linked to increased camalexin accumulation and altered powdery mildew resistance [58]. In soybean hairy roots, targeted manipulation of stilbene or glyceollin production changed chemical and infection readouts [37]. Changes in metabolite pools do not, however, quantify de novo biosynthetic flux unless isotope-based or otherwise direct rate measurements are also performed.

## 7. Spatial Analysis: What Existing Methods Can Test

### 7.1. Published Imaging Precedents and Chemical Specificity

Where a defensive metabolite accumulates is not necessarily where it is synthesised. Mass spectrometry imaging (MSI) measures spatially distributed ions; assignment to a specific structure depends on separation from isomers, adducts and in-source fragments. Grapevine imaging studies have highlighted such ambiguities for resveratrol-related compounds and mapped infection-associated stilbenoid signals under validated analytical conditions [59,60]. Matrix-free ultraviolet laser desorption/ionisation followed by laser postionisation (UV LDI–LPI) has been applied to other plant metabolites [61]. These precedents illustrate distinct analytical capabilities, but no cited primary investigation has provided a chemically authenticated MSI map of debneyol in *Nicotiana* or established the site of its biosynthesis [9,59,60,61].

Because the published UV LDI–LPI work was evaluated with other analytes and matrices, its performance cannot be assumed to apply to debneyol [61]. Nor can a plausible *m*/*z* feature be interpreted as an anatomical map without compound-specific validation. In an infected leaf, a spatial signal may reflect synthesis, movement, binding or degradation; the imaging result alone does not discriminate among these possibilities. This is precisely why a realistic experimental proposal should not be illustrated as though it were an acquired mass spectrum or infection-front map.

### 7.2. A Staged Test of Chemical Identity and Localisation

A credible debneyol imaging programme would begin with an authentic chemical standard and chromatographic or tandem mass spectrometric identification, followed by controls for isomeric compounds, adducts, fragmentation, matrix effects and analyte redistribution during sample preparation. Only after signal-to-background, spatial resolution, and ionisation response have been established should matched infected tissues from appropriate genotypes be compared [59,60,61]. Where putative accumulation domains are detected, orthogonal liquid chromatography–tandem mass spectrometry and time-resolved sampling would help substantiate chemical identity. Distinguishing local biosynthesis from transport or retention would still require genetic, labelling or other independently discriminating experiments [9,10,11,12,59,60,61]. The significance of such measurements depends on their ordering. A genotype-dependent difference in an unverified ion feature is not evidence of metabolic compartmentation. Conversely, a chemically verified spatial gradient would be valuable even if the microscopic transfer of 5-EA remained unresolved, because anatomical accumulation and enzyme-to-enzyme channelling are different biological questions.

## 8. Integrated Evidence and Outstanding Mechanistic Questions

Three distinct levels of mechanistic inference emerge from the available evidence. First, experimentally supported observations include the identification of 5-EA as a shared biosynthetic intermediate, the reconstruction of the EAS–EAE–EH1 sequence leading to debneyol, and miR1919-dependent regulation of MCD1 [7,8,9]. Protein-interaction experiments establish MCD1-associated changes in detectable EAS–EAE interactions, whereas microsomal assays demonstrate increased EAE-dependent catalytic output under defined experimental conditions [9]. Genetic perturbations, metabolite measurements and infection experiments further support the contribution of this regulatory system to plant defence, although they address different levels of biological causality [9].

Second, several mechanistic explanations remain compatible with these observations without having been independently demonstrated. MCD1-dependent protein association could increase the effective availability of 5-EA to EAE, while changes in the proportion of catalytically active EAE, membrane organisation or electron-transfer efficiency could contribute to the measured kinetic response. Altered transcript and enzyme abundance may additionally influence the distribution of debneyol and capsidiol. Direct preferential intermediate transfer is also a possible explanation for enhanced pathway coupling, but the available association and product-formation measurements do not establish protected substrate channelling [9,10,11,12,17,23,24,25,26,27].

Third, important causal alternatives remain experimentally indistinguishable. The current data cannot distinguish between direct EAS-to-EAE intermediate transfer and enhanced conversion following exchange with an accessible 5-EA pool. Similarly, the reported increase in EAE maximum reaction velocity cannot be attributed uniquely to intrinsic catalytic turnover because normalisation to catalytically active enzyme concentration was not established. The respective quantitative contributions of transcriptional regulation, enzyme abundance and protein association to metabolite redistribution also remain unresolved. Furthermore, pathogen-resistance phenotypes cannot be attributed exclusively to the antimicrobial action of debneyol, independent of other MCD1-associated immune and cell-death responses [9].

**Figure 2:**
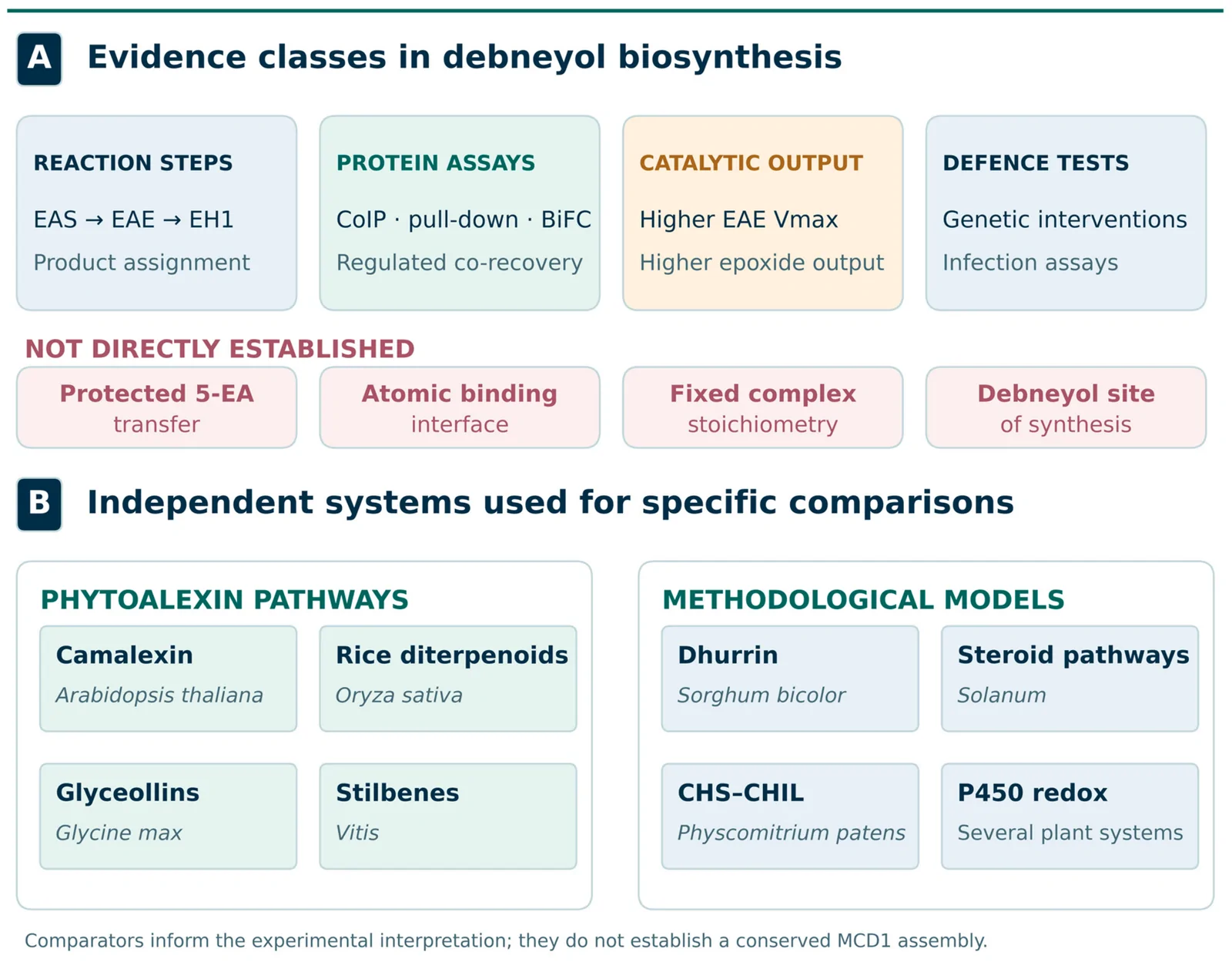
Distinct levels of experimental evidence for miR1919–MCD1-dependent debneyol biosynthesis and chemically independent plant comparisons. (**A**) Reaction chemistry, protein association, and catalytic or infection endpoints are distinguished from mechanisms that have not been directly established in Nicotiana: protected 5-epi-aristolochene (5-EA) transfer, an atomic protein interface, fixed complex stoichiometry, and the anatomical site of debneyol synthesis [9]. (**B**) Camalexin in *Arabidopsis thaliana*, diterpenoid phytoalexins in *Oryza sativa*, glyceollins in *Glycine max* and stilbenes in *Vitis* species illustrate independent phytoalexin chemistry and defence regulation [2,3,4,5,13,18,34,42,43]. Non-phytoalexin methodological comparators include dhurrin in *Sorghum bicolor*, *Solanum* steroidal alkaloids, chalcone synthase–chalcone-isomerase-like (CHS–CHIL) interactions (including *Physcomitrium patens*), and cytochrome P450 redox partners [10,11,14,15,16,17,23,24,25,26,27,46,47,48,49,50]. The CHS–CHIL example refers to a distinct structural and kinetic experimental system, not an established model of MCD1 regulation.

Resolving these questions requires matched experiments that independently vary protein association, enzyme abundance, and intermediate accessibility. Competitive-substrate and isotope-dilution assays could test preferential 5-EA transfer, whereas quantitative measurements of active EAE, membrane-associated electron transfer, transcript abundance and protein stoichiometry could distinguish catalytic from organisational contributions [9,10,11,12,23,24,25,26,27]. Spatially resolved metabolite measurements would provide an additional level of evidence but would not, by themselves, establish microscopic substrate channelling. Figure 2 summarises the distinction between established observations and unresolved mechanisms, while the comparative systems discussed above provide methodological benchmarks rather than evidence for conservation of the MCD1 regulatory architecture [5,10,11,12,13,17].

## 9. Conclusions and Practical Perspectives

The central conclusion of this review is that experimentally demonstrated enzyme organisation must be distinguished from the mechanisms proposed to explain its metabolic consequences. The miR1919–MCD1 module provides compelling evidence that post-transcriptional regulation, protein association and catalytic modulation can converge on phytoalexin biosynthesis. However, these observations represent distinct levels of causal evidence rather than proof of a single integrated mechanism. In particular, functional enzyme cooperation does not establish protected substrate channelling and altered metabolite abundance cannot independently resolve changes in biosynthetic flux or the contribution of competing regulatory processes.

The critical comparison of phytoalexin and non-phytoalexin pathways yields a broader conclusion: plant specialised metabolism is regulated through mechanistically diverse, experimentally distinguishable processes rather than universal metabolon architecture. Evidence from camalexin, rice diterpenoids, glyceollins and stilbenes demonstrates the importance of separating catalytic organisation, transcriptional and small-RNA regulation, pathway chemistry and pathogen-dependent defence. Complementary studies of intermediate accessibility, protein interactions and membrane-associated redox processes establish methodological standards for distinguishing these mechanisms. Their principal value lies not in demonstrating conservation of the MCD1 system, but in defining the limits of cross-system inference.

A further conclusion concerns experimental causality. Protein association, catalytic measurements, transcript abundance, metabolic profiles, and infection phenotypes cannot be treated as interchangeable evidence. Progress will depend on experiments that measure these variables under matched conditions and directly discriminate between competing explanations. For the MCD1 system, the most informative priorities are quantitative assessment of active-enzyme abundance and protein association, controlled tests of intermediate accessibility, and integration of metabolite turnover with spatially and temporally resolved defence responses. Such an approach would establish whether changes in enzyme organisation contribute independently to pathway output and how those effects interact with broader immune regulation.

These conclusions also define the limits of practical translation. Although regulated phytoalexin biosynthesis offers opportunities for engineering chemical defence and developing specialised-metabolite production platforms, neither broad-spectrum field resistance nor general applicability across crop species has been established. Future applications will require species-specific biochemical validation, assessment of resistance under agronomically relevant conditions, evaluation of potential growth and reproductive costs, and demonstration of production feasibility. The value of the miR1919–MCD1 system therefore extends beyond a single biosynthetic pathway: it provides a rigorous experimental framework for evaluating how molecular regulation, enzyme organisation and metabolic defence can be connected without exceeding the evidence available.

## Figures and Tables

**Table 1:** Comparative experimental evidence for plant specialised-metabolite organisation and the limits of cross-system inference. The table compares the debneyol pathway in *Nicotiana* with camalexin, rice diterpenoids, soybean glyceollins, grapevine stilbenes and selected non-phytoalexin metabolic systems. Evidence classes are standardised as biochemical (reaction assignment, pathway reconstruction or intermediate-accessibility experiments), interaction (protein association), kinetic (catalytic measurements), structural (protein structural analysis), genetic (gene perturbation or regulatory interventions), metabolic (metabolite measurements) and phenotypic (infection or resistance outcomes). Each class refers to evidence obtained within the corresponding experimental system. The fourth column identifies transferable analytical principles, whereas the final column specifies mechanistic claims requiring direct validation in *Nicotiana*. Abbreviations: BiFC, bimolecular fluorescence complementation; CHIL, chalcone-isomerase-like protein; CHS, chalcone synthase; CoIP, co-immunoprecipitation; EAE, 5-epi-aristolochene epoxidase; EAS, 5-epi-aristolochene synthase; EH1, epoxide hydrolase 1; GST, glutathione S-transferase; MCD1, miR1919-targeted cell death factor 1.

Experimental System	Evidence Class	Measured Biochemical/Genetic Evidence	Transferable Interpretation	Claim NOT Supported
*Nicotiana* EAS–EAE–EH1–MCD1 (phytoalexin)	Biochemical; interaction; kinetic; genetic; metabolic; phenotypic	Verified EAS–EAE–EH1 chemistry, CoIP, GST pull-down and BiFC, EAE kinetic measurements, product profiling and pathogen endpoints [9].	MCD1-associated enzyme organisation and functional catalytic cooperation.	Protected substrate transfer, sterol-flux control or a resolved EAE allosteric mechanism.
*Arabidopsis* camalexin (phytoalexin)	Biochemical; interaction; kinetic; genetic; phenotypic	P450 pathway reconstruction, protein association, kinetic measurements, genetic perturbations and infection phenotypes [2,13,29,30].	Experimental evidence for catalytic cooperation in a distinct phytoalexin system.	Conserved MCD1 interfaces or universal phytoalexin substrate channelling.
*Oryza* diterpenoid phytoalexins	Genetic; metabolic; phenotypic	OsCPS2/OsCPS4 perturbations, OsWRKY10–OsVQ8 regulation, diterpenoid profiles and pathogen-response measurements [3,18,33].	Genetic and transcriptional regulation can link pathway-specific metabolite changes to pathogen-dependent defence.	An MCD1-like organiser, conserved enzyme assembly or protected intermediate transfer.
*Sorghum* dhurrin (not a phytoalexin)	Biochemical; interaction	Microsomal tests of internally generated versus externally supplied intermediates and reconstitution of a dynamic enzyme assembly [10,11].	Intermediate-accessibility assays provide functional tests of pathway coupling and substrate channelling.	The same enzyme-assembly topology or protected EAS–EAE transfer in *Nicotiana*.
*Solanum* steroidal glycoalkaloids (not phytoalexins)	Biochemical; genetic; metabolic	Enzyme-function assays, genetic perturbations and product analyses defining catalytic and organisational roles [15,16].	Catalytic and organisational functions can coexist in specialised-metabolite pathways.	Functional or structural equivalence of GAME15-related proteins and MCD1.
CHS–CHIL flavonoids (not necessarily phytoalexins)	Biochemical; interaction; kinetic; structural	Interaction assays, partner-dependent product chemistry, structural analysis and kinetic measurements [17,49,50].	Partner-dependent catalytic specificity and molecular dynamics can be tested experimentally.	An analogous MCD1-induced EAE conformational or allosteric mechanism.
Lignin/cytochrome b5 (not phytoalexins)	Biochemical; genetic	P450-associated protein organisation, cytochrome b5 genetics and redox-dependent catalytic assays [14,23,24,25].	Membrane-associated interactions and electron-transfer partners can affect P450 catalytic output.	Direct evidence of cytochrome b5-dependent EAE regulation or substrate channelling by MCD1.
Soybean glyceollins (phytoalexins)	Biochemical; genetic; metabolic; phenotypic	Enzymatic pathway reconstruction, genetic and transcriptional interventions, metabolite analyses and infection phenotypes [4,5,34,35,36].	Biochemical reconstruction and genetic perturbations can independently establish pathway chemistry and its contribution to pathogen-dependent defence.	An MCD1-like organiser, scaffold-dependent glyceollin biosynthesis or protected intermediate transfer.
*Vitis* stilbenes (phytoalexins)	Genetic; metabolic; phenotypic	Stilbene synthase manipulation, miR827–VqMYB14 and miR3398–VqMYB15 regulation, stilbenoid accumulation and resistance-associated outcomes [38,39,40,41,42,43].	Transcriptional and small-RNA-mediated regulation can modify defence-metabolite accumulation and associated infection responses.	miRNA-dependent enzyme recruitment or an MCD1-equivalent metabolon architecture.

## Data Availability

No new data were created or analyzed in this study. Data sharing is not applicable to this article.

## Abbreviations

The following abbreviations are used in this manuscript:

3-Ac-capsidiol	Capsidiol 3-acetate
5-EA	5-epi-aristolochene
5′ RACE	5′ rapid amplification of cDNA ends
BiFC	Bimolecular fluorescence complementation
cDNA	Complementary DNA
CHIL	Chalcone isomerase-like protein
CHS	Chalcone synthase
CoIP	Co-immunoprecipitation
EAE	5-epi-aristolochene epoxidase
EAH	5-epi-aristolochene hydroxylase
EAS	5-epi-aristolochene synthase
EH1	Epoxide hydrolase 1
ER	Endoplasmic reticulum
FPP	Farnesyl diphosphate
GC–MS	Gas chromatography–mass spectrometry
GST	Glutathione S-transferase
MCD1	miR1919-targeted cell death factor 1
miR1919	MicroRNA 1919
miRNA	MicroRNA
mRNA	Messenger RNA
MSI	Mass spectrometry imaging
P450	Cytochrome P450
RNA	Ribonucleic acid
TBF1	TL1-binding factor 1
TNV-AC	Tobacco necrosis virus, AC isolate
uORF	Upstream open reading frame
UV LDI–LPI	Ultraviolet laser desorption/ionisation coupled with laser postionisation
V_max_	Maximum reaction velocity
